# Lesser omental hernia without intestinal gangrene – Case report

**DOI:** 10.1016/j.ijscr.2022.107361

**Published:** 2022-06-27

**Authors:** Arnas Ugianskis

**Affiliations:** Department of Gastrointestinal Surgery, Aalborg University Hospital, Aalborg, Denmark

**Keywords:** CT, computed tomography, GI, gastrointestinal, SMA, superior mesenteric artery, SMV, superior mesenteric vein, BMI, body mass index, Lesser omentum, Hepatogastric ligament, Internal hernia, Colectomy, Lesser sac, Case report

## Abstract

**Introduction:**

There are several types of internal hernia. Herniation through the defect in the lesser omentum is extremely rare. Symptoms of this type of hernias may vary a lot and diagnosis is difficult. In this case report a young adult with nonspecific symptoms is diagnosed with an intestinal herniation through the defect in the lesser omentum.

**Case presentation:**

A 35-year-old man with the history of laparoscopic colectomy presented with abdominal pain but no symptoms of peritonitis or acute bowel obstruction. Abdominal computed tomography (CT) revealed displacement of mesenteric vessels, small intestine and stomach. Intestinal herniation through the lesser omentum was suspected. Laparoscopic reposition of small intestine was performed. The greater curvature of the stomach was sutured to the transverse mesocolon to prevent recurrence of hernia.

**Discussion:**

Previous surgery, low body mass index (BMI), absence of adhesions may predispose the lesser omental hernia. Herniating of intestines through the large openings may occur without presence of acute obstruction or gangrene. CT is helpful in making a correct diagnosis.

**Conclusion:**

When evaluating the patient with abdominal pain, internal hernia should be considered. CT modalities may aid in the detection of these rare hernias and ensure timely treatment. Perioperative inspection and repair of the hepatogastric ligament may help to prevent lesser omental hernias.

## Highlights

• Lesser omental hernia is a rare type of internal hernias with variety of symptoms.
• The diagnosis of lesser omental herniation is challenging.
• Perioperative closure of lesser omental defects may prevent herniation.

## 1 Introduction

Internal hernias are defined by the protrusion of a viscus through a normal or abnormal peritoneal or mesenteric aperture within the peritoneal cavity [1], [2]. These apertures can either be congenital (such as foramen of Winslow, paraduodenal apertures, intersigmoid recess, abnormal apertures arising from incomplete peritoneal rotation) or acquired (traumatic, post-inflammatory or postoperative) [2]. Intestines are the most common contents to herniate.

Internal hernias are uncommon clinical conditions with an overall incidence of less than 1 % whereas the transomental hernias represent 1 to 4 % of all internal hernias with the lesser omental hernias being even rarer [1]. Symptoms vary, are usually nonspecific [3], [4] and diagnosis is difficult. Only few cases of lesser omental hernia have been reported.

We present a case of herniation of small intestine through a defect in the hepatogastric ligament (lesser omentum) without intestinal gangrene. We report this case to enrich a clinical thinking on abdominal pain.

The case-presentation is made in full accordance with the current guidelines for surgical case reports (SCARE) [5].

## 2 Case presentation

A 35-year-old male presented to the emergency department, suffering from postprandial pain in the upper left abdominal quadrant, vomiting and belching for two days. Nine months prior to admission a laparoscopic total colectomy with terminal ileostomy was performed due to refractory ulcerative colitis. The patient had lost four kilograms of body weight over the past week, was dehydrated. He had the body mass index (BMI) of 21. There was no abdominal tenderness or symptoms of peritonitis and acute bowel obstruction was not suspected. The patient had an ileostomy which produced between 2000 and 3000 mL per day.

Laboratory tests revealed a white blood cell count of 20,000/mL, C-reactive protein of 17.0 mg/L, creatinine 181 μmol/L, lactic acid level of 1.2 mmol/L.

A normal upper GI endoscopy was performed, the patient was rehydrated with intravenous fluid and discharged from hospital on day four. Dehydration due to high stoma output was suspected.

The patient was readmitted two weeks later with recurrent symptoms.

**Fig. 1:**
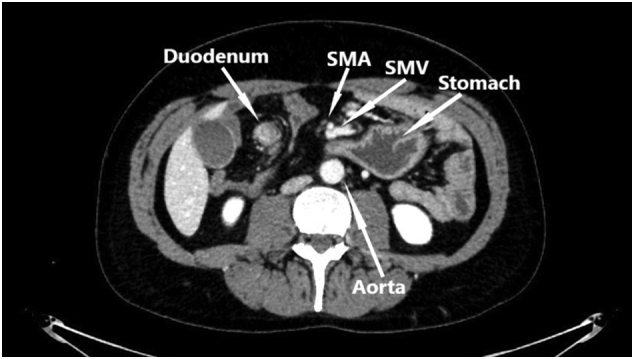
Preoperative contrast enhanced CT study image. Herniation of the mesentery through the lesser omentum.

**Fig. 2:**
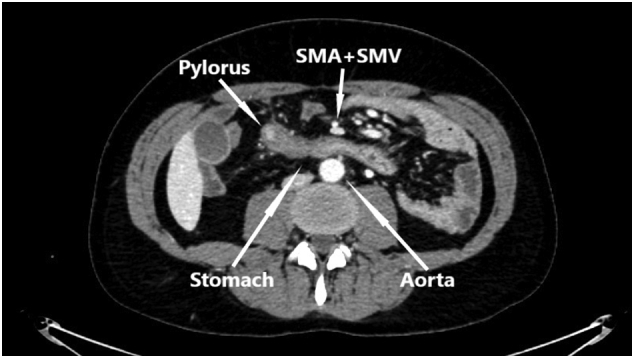
Preoperative contrast enhanced CT study image. Displacement of superior mesenteric artery (SMA) and superior mesenteric vein (SMV) anteriorly to stomach.

**Fig. 3:**
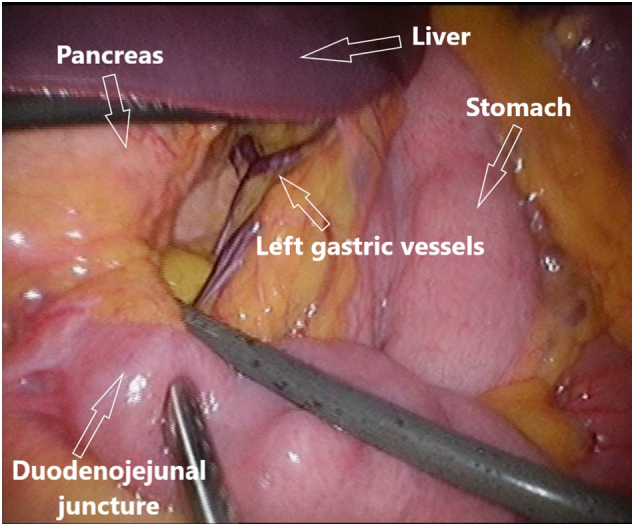
Perioperative laparoscopic still image. Herniation of small intestine and pancreas through the opening in the lesser omentum.

**Fig. 4:**
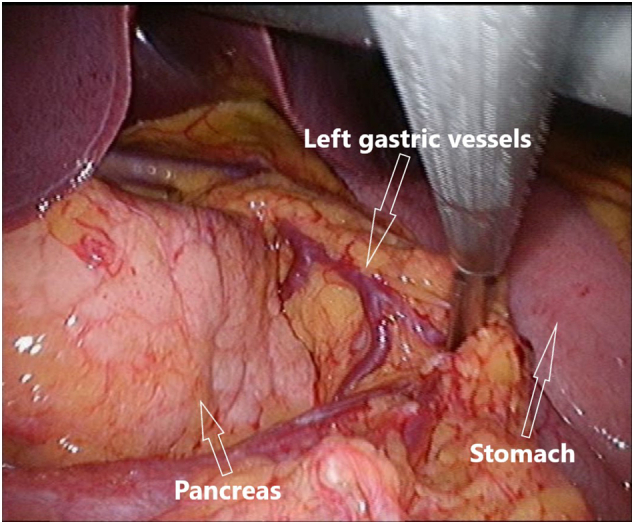
Perioperative laparoscopic still image. A large aperture in the hepatogastric ligament after repositioning of the herniated viscus.

Abdominal computed tomography (CT) showed periduodenal edema but otherwise normal small intestine without signs of mechanical obstruction, intestinal ischemia or intraabdominal liquid accumulation. Common manifestations of internal hernia like swirl sign appearance of the mesenteric vessels or mushroom shape sign was not observed [6]. Due to an elongated J-shaped stomach and the fact that the superior mesenteric vessels were seen in front of the antral part of the stomach on the CT, suspicion of an internal herniation through the hepatogastric ligament without intestinal obstruction was raised (Fig. 1, Fig. 2). Laparoscopy was performed and a complete herniation of small intestine including the duodenojejunal flexure, ascending part of duodenum and a uncinate part of pancreas through the lesser omentum was revealed (Fig. 3). There was no evidence of strangulation. The small intestine was repositioned and a large opening in the hepatogastric ligament was identified (Fig. 4). Because of the size of the opening it was not possible to close the lesser omentum defect sufficiently and tension-free. Instead a neo-lesser sac was created by suturing remnants of the gastrocolic ligament and transverse mesocolon. Suturing was performed with continuous non-absorbable 4-0 polyfilament suture.

Postoperative course was uneventful and the patient was discharged on postoperative day three. He was free of symptoms at one-year follow-up.

## 3 Discussion

Lesser omental hernia is an extremely rare type of internal hernias. Although congenital factors, such as defects of gastrocolic and hepatogastric ligaments can cause this type of internal hernia, lesser omental hernias are most likely to occur in patients with history of abdominal surgery. Roux-en-Y surgery [7], low BMI, decreased amount of visceral fat and a long mesentery may be predisposing factors [8] in addition to fewer or absence of adhesions after laparoscopic surgery [9].

**Fig. 5:**
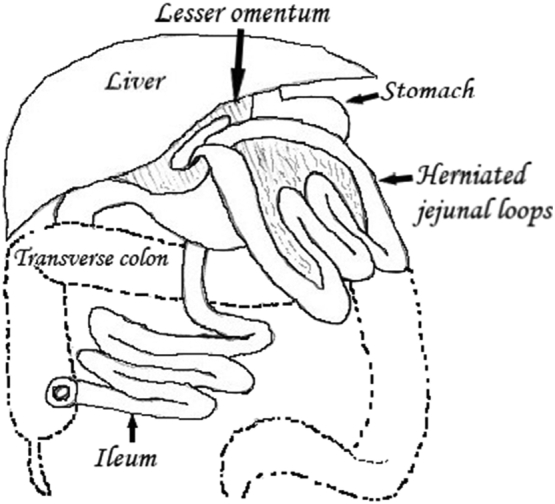
Lesser omental hernia. The large bowel is removed, intestinal loops are able to herniate through the opening in the hepatogastric ligament (lesser omentum).

We have described a retrogastric herniation of the small intestine. The lack of the inferior and partially anterior wall of the lesser sac (transverse mesocolon, transverse colon and the gastrocolic ligament) enabled small intestine to freely prolapse from the retrogastric space through the opening in the hepatogastric ligament (Fig. 5). In the patient with a little of visceral fat almost all mesentery including radix mesenterii was displaced anteriorly to the stomach. Extreme mobility of the intraabdominal viscus in such a slim patient caused the stomach to be displaced caudally. Traction on the mesenteric root caused the fourth part of duodenum and the uncinate process of the pancreas to herniate through the large opening in the hepatogastric ligament (Fig. 3).

The diagnosis of lesser omental herniation is challenging as symptoms may mimic other gastrointestinal diseases [10]. The herniated viscus may resolve spontaneously causing intermittent symptoms or may develop gangrene depending on the size of the defect and length of the herniated bowel loop [3], [4]. Internal hernias often remain undiagnosed prior to emergency surgery as symptoms may be nonspecific and range from mild abdominal discomfort to sudden onset of intestinal obstruction and unexpected death [3], [11], [12].

Contrast enhanced CT may aid in the diagnosis of intraabdominal hernias [13]. Displacement of the superior mesenteric vessels, gathering of mesentery in the lesser curvature of the stomach and distortion of the stomach may be findings that indicate herniation through the lesser omentum.

Lesser omental hernias are most likely to occur in patients with a history of abdominal surgery. In our case the defect in the hepatogastric ligament was most likely caused by previous laparoscopic colectomy. Postoperative lesser omental hernias may be prevented by paying careful attention when dissecting the posterior attachments of the stomach to the transverse mesocolon and by avoiding injury to the hepatogastric ligament. Prophylactic perioperative closure of lesser omental defects may prevent future herniation.

## 4 Conclusions

Although the incidence of the lesser omental hernia is rare, the attention should be paid on the symptoms in the patient with the history of previous surgery. Contrast enhanced abdominal CT may reveal signs of internal hernias, prevent the delay in surgical treatment and avoid complications. Perioperative inspection of the lesser omentum may be effective to avoid iatrogenic intraperitoneal gaps.

## Provenance and peer review

Not commissioned, externally peer-reviewed.

## Consent

Written informed consent was obtained from the patient for publication of this case report and accompanying images. A copy of the written consent is available for review by the Editor-in-Chief of this journal on request.

## Ethical approval

The project is notified and approved by The North Denmark Region Committee on Health Research Ethics.

## Funding

The Department of Gastrointestinal Surgery, 10.13039/501100006304Aalborg University Hospital funded the study. There were no other sources of funding.

## Guarantor

M.D. Arnas Ugianskis, Aalborg University Hospital.

## Research registration number

N/A.

## CRediT authorship contribution statement

AU was the surgeon who performed the laparoscopic colectomy, made clinical case description, compiled literature search and drafted the manuscript.

## Declaration of competing interest

The author has none to declare.
